# Taxonomic and functional nematode diversity in Maldivian coral degradation zones: patterns across reef typologies and depths

**DOI:** 10.7717/peerj.13644

**Published:** 2022-06-30

**Authors:** Eleonora Grassi, Monica Montefalcone, Lucia Cesaroni, Loretta Guidi, Maria Balsamo, Federica Semprucci

**Affiliations:** 1 DiSB, University of Urbino, Urbino, Italy; 2 Fano Marine Center, The Inter-Institute Center for Research on Marine Biodiversity, Resources and Biotechnologies, Fano, Italy; 3 DiSTAV, University of Genova, Genoa, Italy; 4 Consorzio Nazionale Interuniversitario per le Scienze del Mare (CoNISMa), Rome, Italy

**Keywords:** Nematoda, Coral sediments, Functional diversity and redundancy, Human pressure, Reef typology, Maldives

## Abstract

The intensification of dredging and infilling activities in lagoons and on coral reefs are common practices in the Maldivian archipelago, and these activities alter the biodiversity of the bioconstructors and the functioning of the ecosystem. The alteration of environmental factors can also affect inconspicuous fauna, such as free-living nematodes. The implications of a reduction in biodiversity may transcend decreased taxonomic diversity, resulting in changes in functional diversity and redundancy; however, how the environmental conditions and human pressure affects the functionality of nematodes in Maldivian coral degradation zones (CDZs) remain poorly understood. In this paper, we examined changes in the taxonomic and functional diversity and the functional redundancy in nematode communities regarding the geographic location (atolls with various levels of human pressure), the exposure and topography of the reef (lagoon and ocean), the slope of the reef, and the depth. The functional diversity and redundancy were evaluated by considering two main biological traits of nematodes: i) the trophic strategy, and ii) the life strategy. The extremely high number of nematode genera observed in the Maldives is supported by the high complexity of the carbonate sediments. The reef exposure and depth were the most relevant environmental factors that influenced the taxonomic and functional diversity. The functional diversity, according to the trophic strategies, mirrored the taxonomic diversity because the adaptive plasticity of nematode buccal cavity structures is closely associated with the high biodiversity of the phylum. The high abundance of k-strategists in ocean reefs may indicate a higher ecological quality when compared to lagoon reefs; however, the absence of significant differences in life strategy functional diversity and functional redundancy indicates that a recovery process is underway. Analyses of nematode communities should be combined with standard investigations of reef bioconstructors during monitoring activities to assess the vulnerability of CDZ systems to future disturbances and facilitate the adoption of the most appropriate preventative actions.

## Introduction

Coral reef bioconstructions are of particular conservation and scientific interest because they support many microhabitats that promote marine biodiversity (Ape et al., 2018); however, they are declining worldwide due to global and local stressors (Hoegh-Guldberg et al., 2007; Montefalcone, Morri & Bianchi, 2018). In the Maldives, the lack of land and the increasing requirement for private and tourist infrastructures have necessitated the artificial expansion of the islands (Nepote et al., 2016). Therefore, the dredging and infilling of sediment and cement in the surrounding lagoons and coral reefs have been common practices since the 1970s and have intensified since 2005, particularly in the North and South Malé Atolls, which are the most populated areas (Montefalcone, Morri & Bianchi, 2020; Pancrazi et al., 2020). Coastal development, waste disposal and pollution are the primary local human pressures that contribute to the degradation of coral reefs in the Maldivian Archipelago and the alteration of their benthic communities (Pancrazi et al., 2020).

In general, the seabeds associated with reef bioconstructions are dynamic areas that are referred to as coral degradation zones (CDZs) (see Raes et al., 2007), where the sediments constitute the direct remains of corals, green algae (*Halimeda*), coralline red algae, foraminiferans, bryozoans, and molluscs, and are derived from the activity of bioeroders (*e.g*., boring sponges, sea urchins, and parrotfish) (Janßen et al., 2017). The physical disturbance caused by anthropogenic activities (*e.g*., coastal development, bottom trawling, and the disposal of dredged material) may impact seabeds and, consequently, result in changes in invertebrate patterns in coastal systems (Semprucci et al., 2017; Schratzberger & Somerfield, 2020).

Free-living nematodes are essential components of the seabed and provide ecosystem services, including the reworking of the sediments and recycling of organic matter (Schratzberger, Warr & Rogers, 2007). These small metazoans are bound to the seabed throughout their entire lifespan and, therefore, are susceptible to slight environmental alterations. The nematode density and diversity are influenced by the sediment grain size, organic enrichment, salinity, currents, and oxygen availability (Semprucci et al., 2010, and references therein). Natural or human physical disturbances are also important factors that control nematode assemblages in taxonomic and functional terms (Netto, Attrill & Warwick, 1999a; Semprucci et al., 2011; Sandulli, Semprucci & Balsamo, 2014; Semprucci, Colantoni & Balsamo, 2016; Semprucci et al., 2018a; Semprucci et al., 2018b; Maria et al., 2012; Boufahja, Semprucci & Beyrem, 2016). Analyses of meiofauna at higher taxonomic levels are routinely used to detect possible environmental changes (Ape et al., 2018; Losi et al., 2018; Baldrighi et al., 2021). However, the study of nematodes from a taxonomic and functional point of view can contribute more specific information on the ecological status and capacity of ecosystems to respond to disturbances (Semprucci et al., 2011; Losi et al., 2021; Hong et al., 2020; Sahraeian et al., 2020; Schratzberger & Somerfield, 2020).

A high density and biodiversity of nematodes exist in carbonate sediments in the Maldives, where there are an impressive number of microhabitats (Semprucci et al., 2010, 2018a, 2018b). As meiobenthic biodiversity has shown greater vulnerability in lagoon reefs compared to ocean reefs (Semprucci et al., 2018b), in the present study, the composition of nematode assemblages was analysed to detect patterns in taxonomic diversity and community structure across environmental gradients.

Biological diversity not only encompasses a variety of genes, phenotypes, species, etc. but also includes a wide variety of functions that support goods and ecosystem services. Functional groups in nematode communities, *e.g*. trophic guilds (Wieser, 1953) and life strategies (Bongers, Alkemade & Yeates, 1991), can be regarded as clusters of taxa that similarly influence ecosystem processes. According to the redundancy theory, the role of numerous species might be easily taken over by other functionally analogous species without considerably altering ecosystem functioning. Thus, a small number of keystone species may maintain the gains, recovery potential, and stability of an ecosystem after a disturbance (*e.g*., Lawton & Brown, 1993; Kang et al., 2015; Biggs et al., 2020). In this regard, indices that quantify functional diversity and redundancy may help to clarify the relationship between aspects of biodiversity, ecosystem functioning and stability, and environmental constraints, as well as facilitate an assessment of the actual vulnerability of ecosystems (Loreau et al., 2001; Bremner, Rogers & Frid, 2003; Worm et al., 2006).

Most previous studies on the effects of physical disturbances on Maldivian reefs have focused on the response of zooxanthellate scleractinian corals, which generally require long periods to recover (Montefalcone, Morri & Bianchi, 2020; Pancrazi et al., 2020); to date, no information exists regarding the taxonomic and functional changes of benthic nematodes. Hence, in this study, the geographical location of the reef (*i.e*., atolls with various levels of human pressure), the exposure and topography (*i.e*., lagoon and ocean reefs), and the slope and depth of the reef (*i.e*., the main drivers of coral reef zonation Pancrazi et al., 2020) were the environmental factors used to define nematode taxonomic and functional patterns and to quantify changes in functional diversity and redundancy. Four hypotheses were tested, which examined whether the nematode community structure, taxonomic diversity, and functional diversity were influenced by (1) the geographic location in distinct atolls; (2) the exposure; (3) the slope and topography of a reef, and (4) the depth.

## Materials and Methods

### Study area and field activities

**Figure 1:**
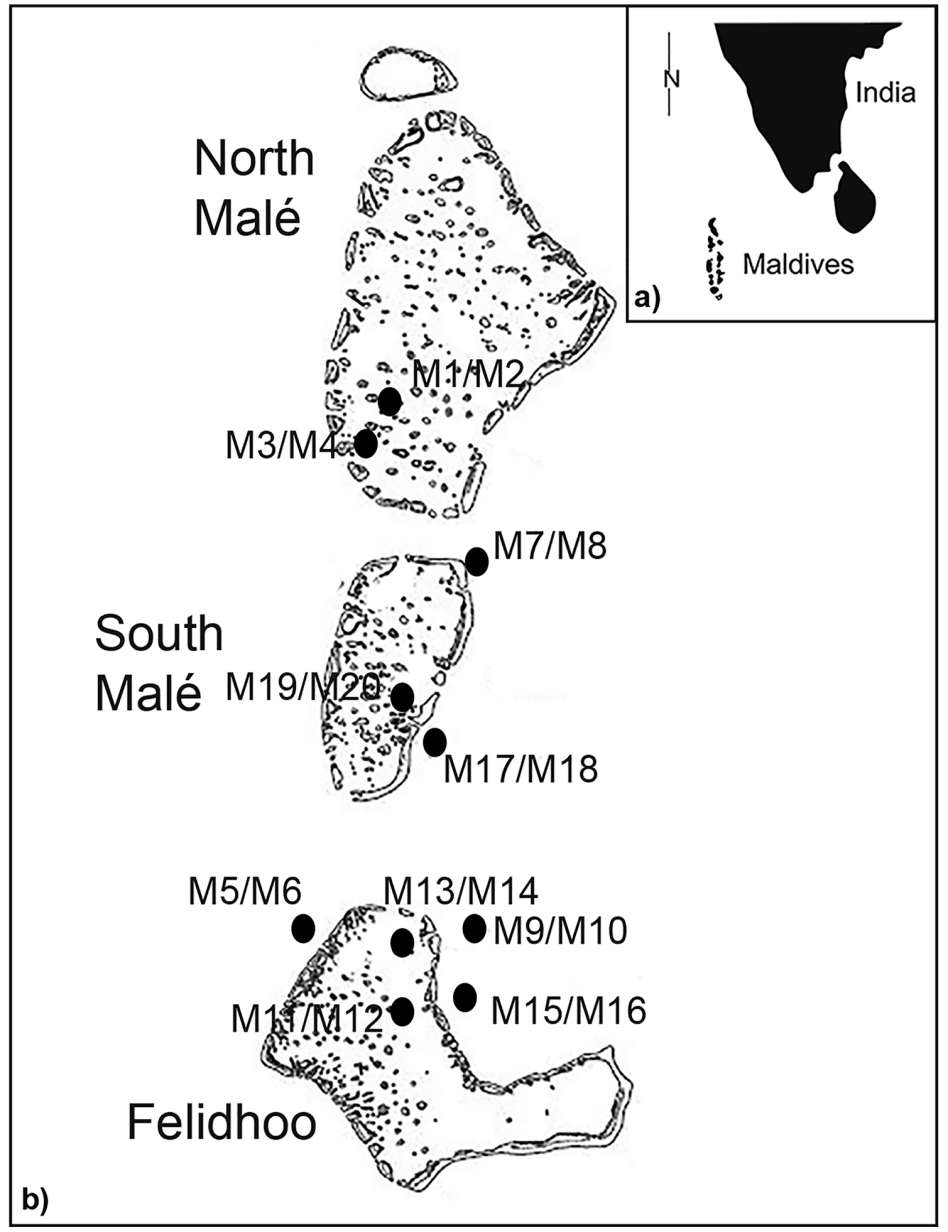
Study area with the locations and codes of the sampling stations (M1 to M20).

The Maldives islands are located in the Indian Ocean, southwest of India (Figs. 1A and 1B). The archipelago occupies a total area of 90,000 km and includes 22 atolls aligned in a north to south direction. The unconsolidated nature of atoll system sediments and seasonally opposing wind directions create an extremely dynamic coastline (Godfrey, 1996; Pancrazi et al., 2020). Atolls have a calcareous nature, and their sediments are derived from the mechanical erosion of coral reefs *via* hydrodynamism and certain bioeroders, such as the parrotfish (Morgan & Kench, 2016). Samples were collected in May 2013 in the atolls of North Malé, South Malé, and Felidhoo. The atolls of North and South Malé have experienced a constant increase in the resident population in recent decades, with a doubling of the population during the past 20 years, whilst the population of Felidhoo decreased slightly during the same period (Montefalcone, Morri & Bianchi, 2020). Additionally, an increase in the number of beds in the resorts (which is a proxy for touristic pressure) was one order of magnitude higher in the atolls of North and South Malé than in Felidhoo (Montefalcone, Morri & Bianchi, 2020).

As reported by Semprucci et al. (2018b), two distinct reef typologies can be distinguished: (i) lagoon reefs, which are the lagoon-facing sides (back reef or inner reef) of the atoll rim or lagoon patch reefs; (ii) ocean reefs, which are the ocean-facing sides (fore reef or outer reef) of the atoll rims. Lagoon reefs have gentle slopes and degrade homogeneously, ending on a sandy terrace at depths no greater than a few tens of metres. Ocean reefs have steep slopes and degrade abruptly from the reef flat to depths greater than one hundred metres (Lasagna et al., 2010) (Figs. 1A and 1B). Erosion processes mainly affect the ocean side of the reef due to its exposure to wind and hydrodynamism. In contrast, the sheltered and calm conditions that characterise a lagoon reef result in faster coral growth and a higher abundance of branching and tabular coral morphologies (Stoddart & Steers, 1977; Morri et al., 2015). Conversely, lagoon reefs are typically more affected by local human pressure (*e.g*., coastal constructions) than ocean reefs (Montefalcone, Morri & Bianchi, 2020; Pancrazi et al., 2020).

In the present study, scuba divers obtained sediment samples at five reef sites in lagoons (inner) and five reef sites on ocean-exposed reefs (outer) (Fig. 1B). At each sampling point, three stations were selected at a depth range from 19 m to 66 m (*i.e*., <25 m, 25–50 m, >50 m) and at the three slopes (*i.e*., <10°, 10–50°, >50°) (Table S1), except for the ocean reefs in which slopes of <10° were absent (total number of stations = 20). The sampling of sediments was conducted using a plexiglass corer (surface area 5.72 cm^2^) that was manually pushed to 5 cm into the sediment. At each station, three replicates of sediments were obtained for the taxonomic analysis of nematodes (Vincx, 1996) and one sediment sample was obtained for sedimentological analysis. A total of sixty samples were analysed for nematodes and twenty samples were examined for granulometry. The samples for nematode analysis were treated with an aqueous solution of magnesium chloride (final concentration 7%) for the narcotisation and relaxation of the meiofaunal organisms. The samples were subsequently treated with a 10% solution of neutralised formaldehyde for fixation (see Danovaro et al., 2004 for details).

### Sedimentological analysis

The diffraction and diffusion of a monochromatic laser beam diffused on suspended sediment particles (Particle Size Analyzer Malvern Mastersizer 2,000) were utilised for the sedimentological analyses according to the protocol described by Loizeau et al. (1994). The measurements ranged from 0.02 to 2,000 μm, with obscuration of 10% to 20%. The sediments were subdivided into the following granulometric classes: clay (particle size: <3.9 μm), silt (from 3.9 to 63 μm), and sand (>63 μm) and utilised for further characterisation of the faunal microhabitat.

### Nematode community structure analysis and ecological indices

In the laboratory, the samples were sieved through a 500 μm mesh to remove macrobenthic organisms, while a 42 μm mesh was utilised to retain meiobenthic nematodes (Danovaro et al., 2004). The sediment fraction from 500 μm and 42 μm was thoroughly rinsed with a gentle jet of prefiltered water. For the extraction of the fauna from the sediment fraction, flotation and multiple decantations (10 times) were performed, followed by three Ludox gradient centrifugations (Ludox HS 30, final density 1.18 g/cm^3^, 3,000 rpm for 10 min) (Boufahja, Semprucci & Beyrem, 2016). The extracted nematodes were preserved in a 10% formaldehyde solution that included several drops of Rose Bengal stain (0.5 g L^−1^, see Danovaro et al., 2004) to facilitate subsequent counting and sorting. A total of 100 specimens were randomly chosen from each replicate under a stereomicroscope (Leica G26, zoom 25×–50×, Semprucci et al., 2010) and transferred into a capsule containing a glycerine solution. The specimens were preserved on permanent slides (Seinhorst, 1959) and identified at the genus level under a light optical microscope (Nikon Optiphoto-2) using taxonomic guides and original species descriptions available on the Nemys website (Bezerra et al., 2021).

The Shannon-diversity (H’) and Pielou-evenness (J) (both log2) indices were computed to provide a measure of the taxonomical diversity of the nematode community. To explore the functional structure of the community, the nematode genera were classified according to two main biological traits: i) the trophic strategy, and ii) the life strategy. Four trophic guilds were identified based on the structure of nematode buccal cavities (Wieser, 1953): selective and non-selective deposit feeders (1A and 1B, respectively), epigrowth feeders (2A), and omnivores/carnivores (2B). Five life strategies were defined according to the five coloniser-persister (c-p) classes distinguished by Bongers, Alkemade & Yeates (1991), in a spectrum from extremely tolerant species (*i.e*., extreme r-strategists or c-p 1: short generation time, high egg production, high colonisation ability, and tolerance to stress) to extremely sensitive nematodes (*i.e*., k-strategists or c-p 5: long generation time, very few offspring, low colonisation ability, and sensitivity to stress). The four trophic guilds and the five life strategies were utilised to measure the functional diversity and the functional redundancy (Lawton & Brown, 1993; Petchey & Gaston, 2006), using the Shannon-diversity (H’) and the Simpson-dominance (D) indices, respectively.

### Data analysis

All the nematode taxa identified were utilised to create a data matrix for the following statistical analyses. Non-metric multi-dimensional scaling (nMDS) analysis, derived from Bray–Curtis similarity matrices (fourth root transformed values), was used to visualise differences in the taxonomical diversity of nematode communities considering the following factors: (i) the geographic location (the three atolls); (ii) the exposure (lagoon and ocean reefs); (iii) the slope (*i.e*. <10°, 10–50°, >50°), and (iv) the depth (*i.e*. <25 m, 25–50 m, >50 m). The stress values of nMDS can be regarded as a measure of the goodness-of-fit of the ordination, and values lower than 0.2 underline limited probabilities of misleading interpretations (Clarke & Warwick, 1994). Significant differences in the four factors were further examined using analysis of similarities (ANOSIM). A similarity percentage (SIMPER) procedure (cut-off 50%) was performed on the transformed Bray–Curtis similarity matrix to assess the percentage dissimilarity in nematode composition in the four factors. The same multivariate analyses were performed on untransformed data to evaluate differences in the nematode functional traits considering the four factors (Semprucci et al., 2018a).

The Kruskal–Wallis test was applied to evaluate changes in the taxonomic and functional diversity values (*i.e*., Shannon-diversity, Pielou-evenness, Simpson-dominance), trophic guilds (*i.e*., 1A, 1B, 2A, 2B) and life strategies (c-p classes). The software package Primer v.6 (Clarke & Gorley, 2006) was utilised for all the multivariate analyses and computation of the indices (*i.e*., Shannon, Pielou, and Simpson), while the SPSS v.21 software package was used for the Kruskal–Wallis test.

## Results

Calcium carbonate was the dominant component in the sediments (98%). Silt and sand were the prevalent sediment fractions (sand ranged from 20% to 70%, silt from 29% to 73%), while clay was an extremely small component (less than 7.5%) (Table S1).

A total of 173 genera and 36 families were observed in the study area. The families Desmodoridae (26 genera), Chromadoridae (19 genera), Xyalidae (13 genera), and Cyatholaimidae (12 genera) showed the highest richness of genera (Table S2). The Desmodoridae family represented 37% of the entire community, followed by Chromadoridae (11%), Xyalidae (5%), and Cyatholaimidae (5%).

**Figure 2:**
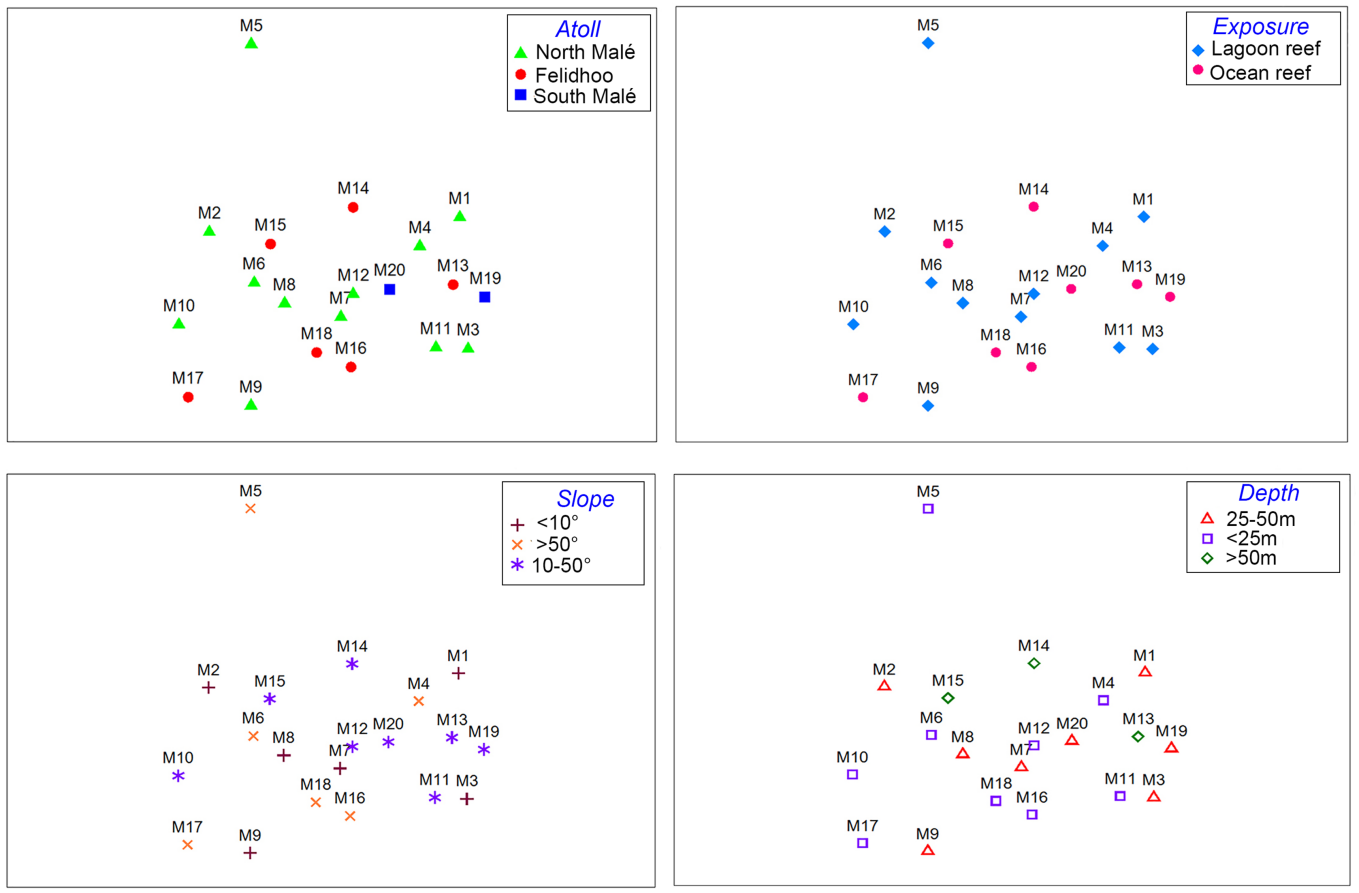
Non-metric multi-dimensional scaling (nMDS, stress value = 0.17) performed on the nematode community structure (fourth root transformed values) considering the four environmental factors (*i.e*. atoll, exposure, slope, and depth).

**Figure 3:**
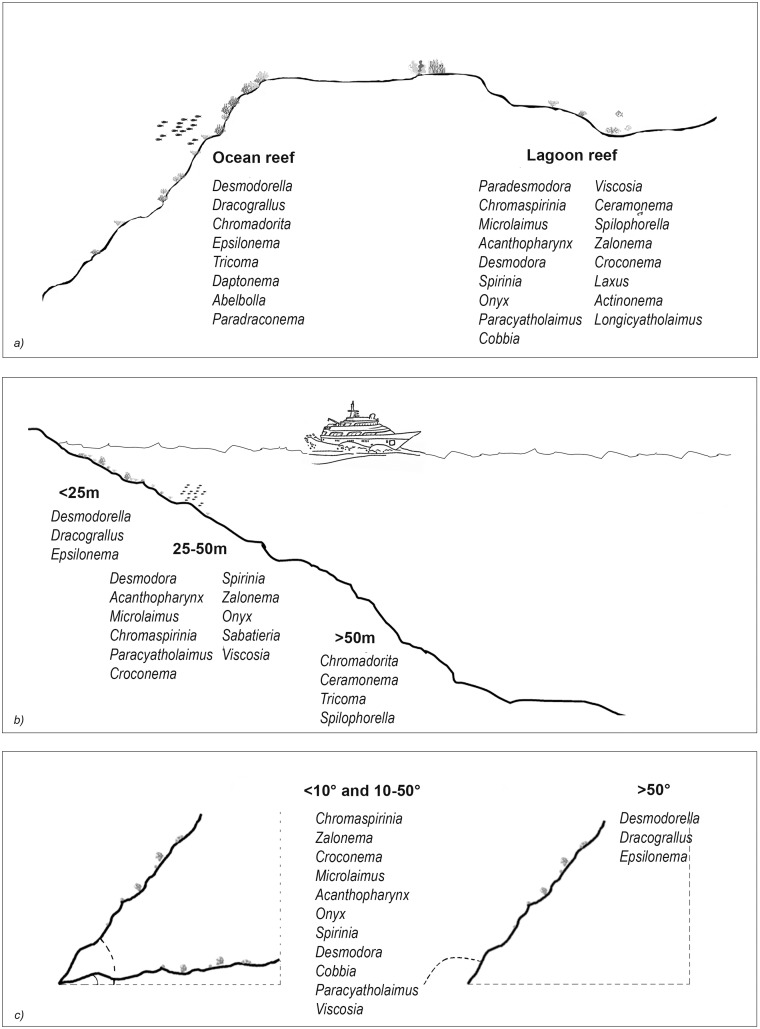
The nematode genera that were important for differentiating reef typologies (A), depths (B), and slopes (C), as obtained by SIMPER analysis (cut-off 50%).

The results of the nMDS (Fig. 2) and ANOSIM (Table 1) on the nematode community structure showed significant differences between the lagoon and ocean reefs (*p* < 0.001), the slope (*p* < 0.01), and the depth (*p* < 0.001), but not between the atolls (*p* > 0.05). The lagoon reefs revealed a higher number of genera compared with the ocean reefs (regarding the total number of genera and the SIMPER results) and mainly consisted of the families Desmodoridae, Microlaimidae, Cyatholaimidae, and Chromadoridae (Fig. 3A; Tables S2 and S3). The Desmodoridae were also observed in ocean reefs, but higher densities of Draconematidae, Epsilonematidae, and Desmoscolecidae were detected in these locations. Differences in the composition of the taxa with depth were observed, with the genera *Desmodorella*, *Dracograllus*, and *Epsilonema* mainly characterising depths less than 25 m; genera of the family Desmodoridae (*i.e*., *Desmodora*, *Acanthopharynx*, *Chromaspirinia*, *Croconema*, *Spirinia*, *Zalonema*, and *Onyx*) as well as *Microlaimus*, *Paracyatholaimus*, *Sabateria*, and *Viscosia* characterising the intermediate depths (25–50 m), while *Ceramonema*, *Tricoma*, *Chromadorita*, and *Spilophorella* were dominant at the deepest stations below 50 m (Fig. 3B; Table S4). Slopes greater than 50° were observed at shallower depths and were characterised by *Desmodorella*, *Dracograllus*, and *Epsilonema*. A significant difference was observed between slopes <10° and slopes between 10° and 50° as well as between slopes of <10° and >50°, which were characterised by the genera that were more abundant at depths between 25 and 50 m (Figs. 3B and 3C; Table S5).

**Table 1:** Analysis of Similarities (ANOSIM) to detect possible differences in nematode community structure among the four environmental factors.

Factors	R	*p*	*Pair-wise* test
Atoll		n.s.	–
Exposure	0.24	<0.001	–
Slope	0.15	<0.01	<10° *vs*. 10°–50° (R = 0.18; *p* < 0.01); <10° *vs*. >50° (R = 0.20; *p* < 0.01); >50° *vs*. 10°–50°(n.s.)
Depth	0.23	<0.001	25–50 m *vs*. >50 m (R = 0.44; *p* < 0.001); <25 m *vs*. >50 m (R = 0.25; *p* < 0.04); 25–50 m *vs*. <25 m (R = 0.13; *p* < 0.05)

**Note:**

n.s., not significant differences; R, ratio that compares the mean of ranked dissimilarities between groups to the mean of ranked dissimilarities within groups; p, probability level.

**Figure 4:**
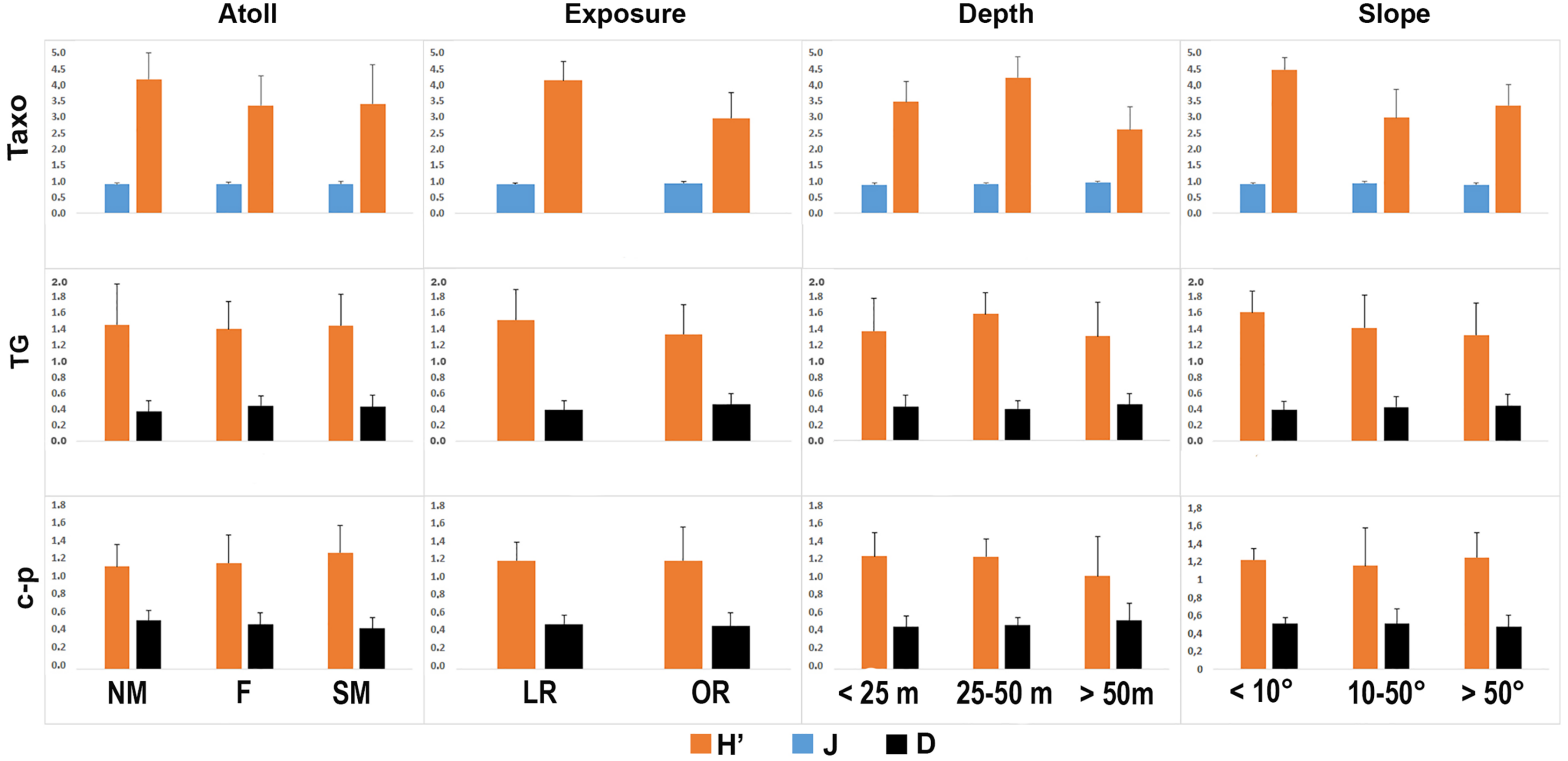
Values of the taxonomic (H’ and J) and functional (H’) diversity and the functional redundancy (D) indices in the nematode community according to the four environmental factors considered (*i.e*. atoll, exposure, depth, and slope). TAXO, taxonomic composition at the genus level; TG, trophic guilds; c-p, life strategies.

**Figure 5:**
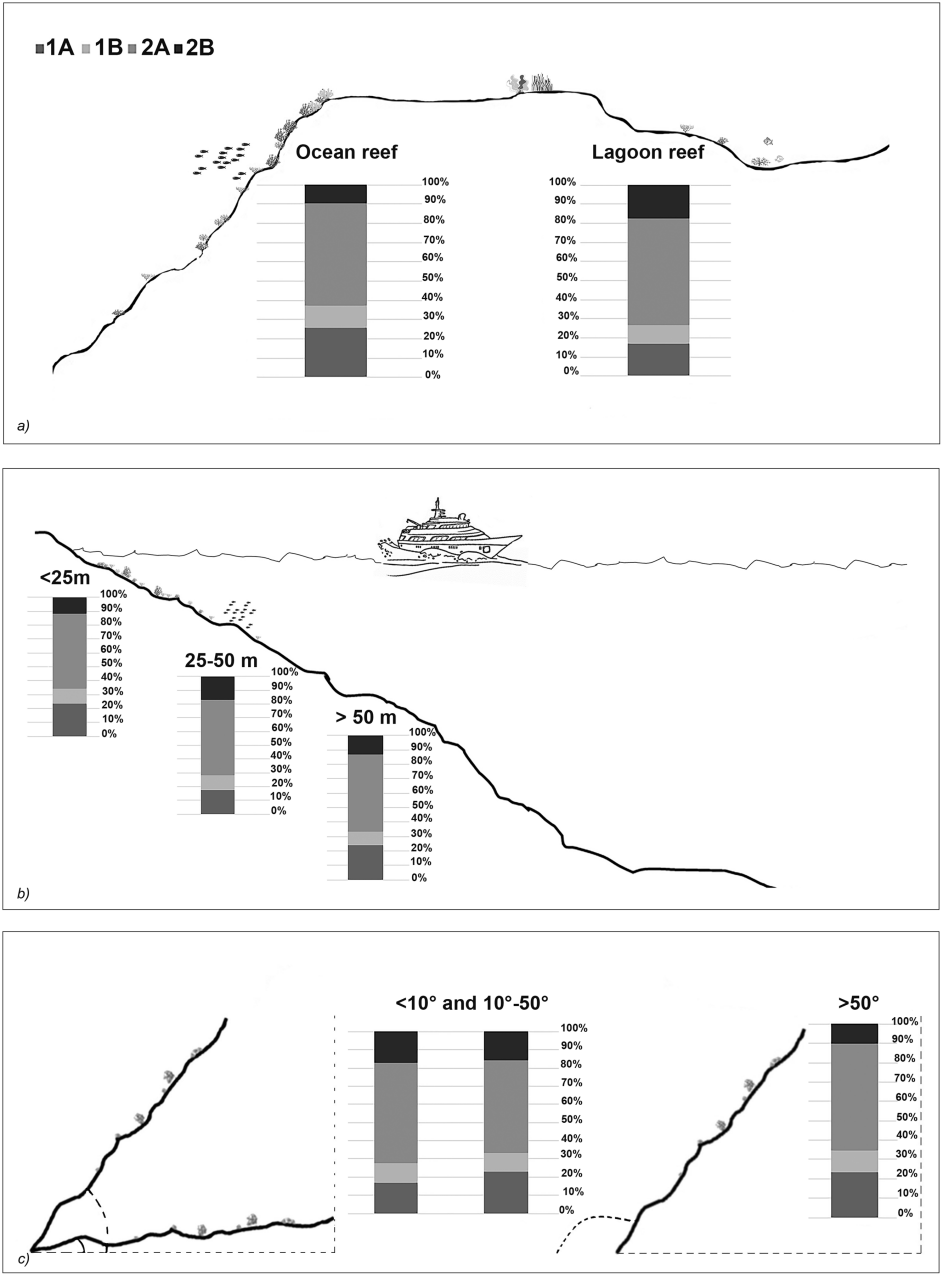
Nematode trophic guilds (*i.e*. 1A, 1B, 2A and 2B) that characterized each reef typology (A), depth (B), and slope (C).

**Figure 6:**
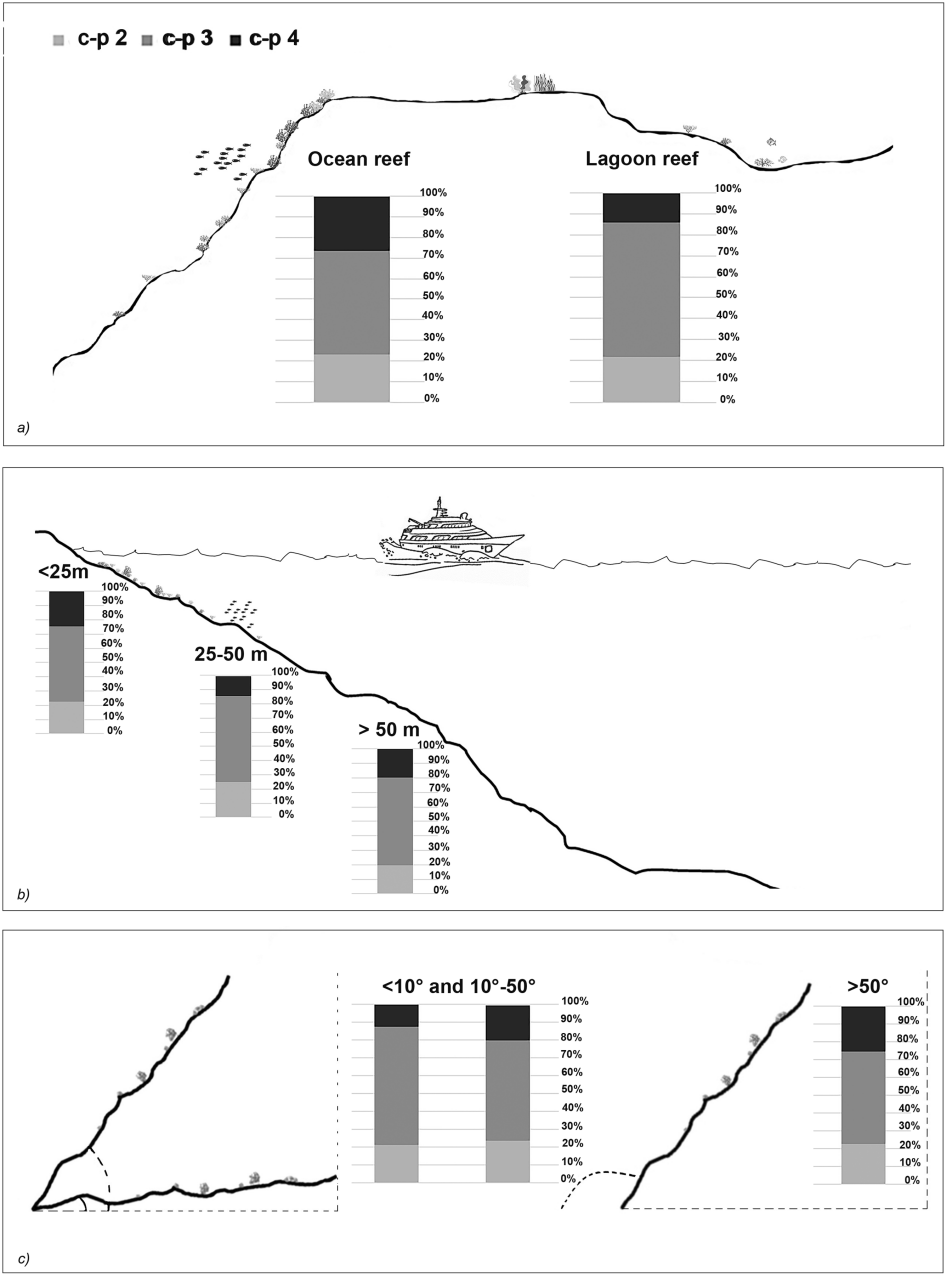
The nematode life strategies (*i.e*. c-p 2, c-p 3, c-p 4) that characterised each reef typology (A), depth (B), and slope (C).

The taxonomic and functional diversity values, as well as the functional redundancy values, are shown in Fig. 4. The number of genera (S) and the Shannon index (H’) were significantly higher in North Malé, while Felidhoo showed the lowest values (*p* < 0.05). The S, H’, and J indices revealed significant differences according to the reef typology, slope, and depth (Kruskal–Wallis test, S: *p* < 0.001; H’: *p* < 0.001; J: *p* < 0.01). S and H’ were higher in the lagoon reefs, while J was higher in the ocean reefs. The highest values of S and H’ were observed at depths of 25 to 50 m and slopes of less than 10°, while the highest values of J were observed at depths greater than 50 m and slopes between 10° and 50°. No significant changes in the functional redundancy among the factors were observed, while a significant difference was observed in the trophic functional diversity concerning the reef typology (*p* < 0.05), with lower values detected for the ocean reefs. The nematode community was mostly characterised by the trophic category 2A (grazers), which represented 54% of the total number. Selective deposivores (1A) represented 22% of the total, predators-omnivorous (2B) represented 14% of the total, and non-selective deposivores (1B) represented 11% of the total (Figs. 5A–5C). A total of 57% of the nematode community was represented by the c-p 3 life strategy, 22% were c-p 2, 20% were c-p 4, and c-p 5 and c-p 1 were only sporadically observed (0.2% and 0.1%, respectively) (Fig. 6).

The ANOSIM showed a significant change in the functional structure (*i.e*., the trophic and life strategies) only between the lagoon and ocean reefs (R = 0.13, *p* < 0.001). The Kruskal–Wallis test also revealed significant differences among the exposure due to trophic groups such as 1A (*p* < 0.05) and 2B (*p* < 0.01), as well as the life strategy categories such as c-p 3 (*p* < 0.05) and c-p 4 (*p* < 0.01). Significant differences were only observed for the c-p 5 life strategy (*p* < 0.001) regarding the depth, whereas significant differences were only observed for the c-p 3 life strategy concerning the slopes (*p* < 0.05) (Figs. 5A–5C and 6).

## Discussion

The richness of the nematode genera recorded in the present study was notably high in comparison with data available from shallow and deep subtidal soft-bottom habitats in other Maldivian localities (Semprucci et al., 2010; Semprucci et al., 2011; Semprucci et al., 2013), in Kenya and Zanzibar (Raes et al., 2007), and the Porcupine Seabight (Raes & Vanreusel, 2006). The Desmodoridae, Chromadoridae, and Xyalidae were the most abundant nematode families, and species belonging to these families have been widely documented in Maldivian CDZs, from shallow to deep seabeds (Semprucci et al., 2010; Semprucci et al., 2011; Semprucci et al., 2013). The Desmodoridae and Chromadoridae are typical components of medium-coarse sediments and CDZs (*e.g*., Raes et al., 2007; Armenteros et al., 2019; Neres, Dos Santos & Esteves, 2021). The Xyalidae, although often associated with fine-grained sediments, includes several species that are associated with coarser sediments (Venekey et al., 2014).

While higher values for the taxonomic richness and the Shannon index were observed at the North Malé atoll, no significant differences were observed in the nematode community structure among the atolls, *i.e*. among various levels of human pressure, even when the atolls were geomorphologically different or located far from each other. Previous investigations in the Felidhoo and South Malé atolls revealed similar results (Semprucci et al., 2010). The numerous cosmopolitan meiobenthic species (‘Everything is Everywhere’ paradox) and a low dissimilarity in the nematode community structure over large spatial scales are not surprising findings and are likely associated with active or passive migration and phoresis phenomena (Baldrighi et al., 2020; Ingels et al., 2020). Instead, a plethora of micro-variations in the physical-chemical environmental features (*e.g*., granulometry, biogeochemistry, food availability, and sediment oxygenation) can significantly control small-scale spatial variations of meiobenthic organisms (Semprucci et al., 2010).

A greater influence of exposure and depth was previously detected in meiofauna applying the surrogacy at major taxon level (Semprucci et al., 2018b). These natural environmental factors likely play a more predominant role in nematode communities than human influence. The dissipation of wave energy across the reef front generally results in different hydrodynamic conditions that lead to the creation of distinct environmental zonations (Lasagna et al., 2010). Lagoon and ocean reefs show distinct coral community composition and functioning, and coral growth, vitality and recruitment rate (Cardini et al., 2012; Jimenez et al., 2012; Nepote et al., 2016; Montefalcone, Morri & Bianchi, 2018) that support parallel shifts in the associated nematodes. The microhabitat structure, rather than hydrodynamism, seems to influence to a larger extent the nematode community composition. Living corals and coral rubble, after mechanical and bio-erosion, may supply seabeds with complex structures and provide extra surfaces for attachment and refuge, sediment or organic matter traps, and food items (*e.g*., bacteria and microalgae) for nematodes (Ape et al., 2018). In Indonesia, changes in the grain size distribution and composition of sedimentary facies between inner and outer reefs, due to a different proportion of coral fragments, shells, and hard parts of reef-forming and reef-associated calcifying organisms, have been documented (Janßen et al., 2017).

The nematode assemblages that inhabited the two reef typologies were dissimilar, with a predominance of Desmodoridae (including Stilbonematinae) and Microlaimidae in the lagoon reefs, while Desmodoridae, Draconematidae, Epsilonematidae, and Desmoscolecidae were predominant in the ocean reefs. The Desmodoridae are the most ubiquitous and abundant nematode family that inhabit carbonate sediments (*e.g*., see De Troch et al., 2008; Semprucci et al., 2010 for review), therefore, itself this family cannot be considered as discriminant of a particular ecological condition. Conversely, genera of the Desmodoridae family varied among the various environmental factors. Representatives of the Stilbonematinae subfamily were consistently observed in the lagoon reefs. This taxon occurs globally in sheltered intertidal and subtidal habitats and is frequently observed in tropical carbonate sediments (Semprucci et al., 2010; Armenteros, Ruiz-Abierno & Decraemer, 2014). Species of this subfamily show an ectosymbiosis with sulphur oxidising bacteria, which cover the cuticle of the nematodes and are often genus- and species-specific consortia (Bayer et al., 2009; Scharhauser et al., 2020). As sediment-dwellers, the Stilbonematinae migrate up and down through the redox cline and, thus, provide the bacteria with electron donors and electron acceptors, such as nitrate and oxygen (Hentschel et al., 1999; Bayer et al., 2009). The exclusive occurrence of members of the Stilbonematinae with many species in the lagoon reefs is likely associated with low hydrodynamism.

Members of the Draconematidae and Epsilonematidae families are morphologically unusual and specialised and are considered epifaunal organisms in the coral fragments of the CDZs (Raes & Vanreusel, 2006; Raes et al., 2007). They have small and sturdy bodies, cuticle annules lined with spiny ornamentation, many adhesive tubes, and a distinctive type of locomotion–looper-like crawling, as is usual in Geometridae worms (Lepidoptera). These organisms are, therefore, capable of moving across various types of substrata, from gravel to coarse sands to smooth coral surfaces or sponge fragments, where other nematode species have difficulty finding a grip (Gad, 2009). Such taxa that have adapted to crawl on larger surfaces and withstand hydrodynamically active environments may have a competitive advantage in ocean reefs (Raes & Vanreusel, 2006; Raes, Decraemer & Vanreusel, 2008; Semprucci et al., 2011). The Desmoscolecidae was an additional family that differentiated reef typologies and its members were more abundant in the ocean reefs at the greatest depths. Species of the family Desmoscolecidae have been defined as ‘sediment-dwelling taxa’, *i.e*., organisms that inhabit the finest coral sands trapped between larger sponge and coral fragments (Raes & Vanreusel, 2006; Semprucci et al., 2013). They show an annulated cuticle covered with desmens (*i.e*. concretion annules made of sedimentary material). Depth was the second environmental factor that accounted for significant differences in the nematode communities and their taxonomic diversity and evenness, with the overall highest values observed at depths greater than 25 m. The Desmoscolecidae were more abundant at depths greater than 50 m, as previously reported from other deep-sea habitats, such as abyssal plains and continental slopes (*e.g*., Zeppilli et al., 2014; Sergeeva & Revkova, 2021). Members of the Ceramonematidae family were also observed at the greatest depths. This family is often associated with the gravelly sediments of seamounts; the species are generally long with a coarsely annulated and extremely flexible cuticle and body, which may be highly advantageous in biogenic sands (Vanreusel et al., 2010).

The reef slope was less relevant for nematodes and the slight differences observed reflect the influence of the depth. Slopes of less than 10° were exclusively associated with certain depths, and it may be difficult to discriminate the effects of these two factors.

The feeding guilds differed between the lagoon and ocean reefs due to the significant contribution of selective deposivores (1A). These organisms have extremely small buccal cavities, thus, they feed selectively on small food items (*e.g*., bacteria; Wieser, 1953). Despite the highest hydrodynamism, bacteria are more abundant in the ocean reefs (Ndaro & Olafsson, 1999; Semprucci et al., 2010; Semprucci et al., 2011). The predators/omnivores (2B) were more abundant in the lagoon reefs. In the Maldives, this guild is associated with medium sands where there is a higher abundance of prey represented by other meiofaunal organisms or macrofaunal juveniles (Netto, Warwick & Attrill, 1999b; Semprucci et al., 2018b). The epigrowth feeders (2A) did not vary between the environmental factors. The high preponderance of this trophic strategy (mostly represented by the Desmodoridae) in carbonate sediments is associated with the considerable level of primary production in CDZs, where these species obtain high densities of diatoms or microalgae (Raes et al., 2007). Moreover, the 2A feeders can be viewed as an opportunistic group that can also benefit from bacterial biofilms that settle in the porosities of biogenic sediments.

Furthermore, the trophic functional diversity mirrored the taxonomic diversity due to the large trophic spectrum and the high adaptive plasticity of nematode buccal cavity morphologies that are closely associated with the high biodiversity of the phylum (De Meester et al., 2016; Daly et al., 2021). Thus, a reduction in the functional diversity could, in the future, be considered an indirect measure of biodiversity loss.

The life strategy composition revealed a medium-high ecological quality in the study area. A higher percentage of c-p 4 in the ocean reefs suggests that the impact was lower here, while inside the lagoon, where a greater proportion of human activity is concentrated, the significant preponderance of intermediate colonisers (c-p 3) indicates recovery. This result, in addition to the absence of significant differences in the life strategy functional diversity and functional redundancy between the lagoon and ocean reefs, suggests higher resilience and recovery capability of the nematodes (Semprucci, Colantoni & Balsamo, 2016) compared to other biological components (*e.g*., corals) regarding physical disturbances.

## Conclusions

Understanding the effects of anthropogenic impacts on tropical regions is extremely important, as these geographic areas are experiencing significant environmental changes. The nematode community of the Maldivian coral reefs investigated during the present study revealed high biodiversity and overall good ecological quality. The nematode life strategies suggested the higher ecological quality of the ocean reefs and signs of community recovery in the lagoons. However, the results suggest that the taxonomic and functional structure of the nematodes is mostly guided by natural environmental conditions and not anthropogenic pressure. Thus, this study confirms the requirement to include small invertebrates, such as nematodes, in the biomonitoring activities of CDZs because these species may contribute to an assessment of the vulnerability and resilience of the benthos. Based on the results of the present study, an assessment of the natural environmental characteristics, together with the nematode structure, is recommended for inclusion in ecological impact assessments on coral reefs.

## Supplemental Information

10.7717/peerj.13644/supp-1 Supplemental Information 1 Study stations: location, atoll, depth, geographic coordinates and inclination of each station.

10.7717/peerj.13644/supp-2 Supplemental Information 2 Presence/absence of families and genera along with the overall number of taxa found for sampling stations.

10.7717/peerj.13644/supp-3 Supplemental Information 3 Results of SIMPER test assessing the dissimilarities in the nematode assemblage structure between the two exposures.

10.7717/peerj.13644/supp-4 Supplemental Information 4 Results of SIMPER test assessing the dissimilarities in the nematode assemblage structure between depths *i.e*. <25 m, 25–50 m, >50 m.

10.7717/peerj.13644/supp-5 Supplemental Information 5 Results of SIMPER test assessing the dissimilarities in the nematode assemblage structure between slope inclinations.

## Additional Information and Declarations

### Data Availability

The following information was supplied regarding data availability: The raw measurements are available in the Supplemental Files.

## References

[ref-1] Ape F, Gristina M, Chemello R, Sarà G, Mirto S (2018). Meiofauna associated with vermetid reefs: the role of macroalgae in increasing habitat size and complexity. Coral Reefs.

[ref-2] Armenteros M, Pérez-García JA, Marzo-Pérez D, Rodríguez-García P (2019). The influential role of the habitat on the diversity patterns of free-living aquatic nematode assemblages in the cuban archipelago. Diversity.

[ref-3] Armenteros M, Ruiz-Abierno A, Decraemer W (2014). Taxonomy of stilbonematinae (nematoda: desmodoridae): description of two new and three known species and phylogenetic relationships within the family. Zoological Journal of the Linnean Society.

[ref-4] Baldrighi E, Dovgal I, Zeppilli D, Abibulaeva A, Michelet C, Michaud E, Franzo A, Grassi E, Cesaroni L, Guidi L, Balsamo M, Sandulli R, Semprucci F (2020). The cost for biodiversity: records of ciliate-nematode epibiosis with the description of three new suctorian species. Diversity.

[ref-5] Baldrighi E, Vasapollo C, Grassi E, Alvisi F, Cesaroni L, Balsamo M, Semprucci F (2021). Meiobenthic assemblages as ecological indicator of natural variability induced by ecosystem engineers. Regional Studies in Marine Science.

[ref-6] Bayer C, Heindl NR, Rinke C, Lücker S, Ott JA, Bulgheresi S (2009). Molecular characterization of the symbionts associated with marine nematodes of the genus Robbea. Environmental Microbiology Reports.

[ref-7] Bezerra TN, Eisendle U, Hodda M, Holovachov O, Leduc D, Mokievsky V, Peña Santiago R, Sharma J, Smol N, Tchesunov A, Venekey V, Zhao Z, Vanreusel A (2021). Nemys: world database of nematodes. http://nemys.ugent.be.

[ref-8] Biggs CR, Yeager LA, Bolser DG, Bonsell C, Dichiera AM, Hou Z, Keyser SR, Khursigara AJ, Lu K, Muth AF, Negrete B, Erisman BE (2020). Does functional redundancy affect ecological stability and resilience? A review and meta-analysis. Ecosphere.

[ref-9] Bongers T, Alkemade R, Yeates GW (1991). Interpretation of disturbance-induced maturity decrease in marine nematode assemblages by means of the maturity index. Marine Ecology Progress Series.

[ref-10] Boufahja F, Semprucci F, Beyrem H (2016). An experimental protocol to select nematode species from an entire community using progressive sedimentary enrichment. Ecological Indicators.

[ref-11] Bremner J, Rogers SI, Frid CLJ (2003). Assessing functional diversity in marine benthic ecosystems: a comparison of approaches. Marine Ecology Progress Series.

[ref-12] Cardini U, Chiantore M, Lasagna R, Morri C, Bianchi CN (2012). Size-structure patterns of juvenile hard corals in the Maldives. Journal of the Marine Biological Association of the United Kingdom.

[ref-13] Clarke KR, Gorley RN (2006). PRIMER v6: user Manual/Tutorial.

[ref-14] Clarke KR, Warwick RM (1994). Changes in marine communities: an approach to statistical analyses and interpretation.

[ref-15] Daly AJ, De Meester N, Baetens JM, Moens T, De Baets B (2021). Untangling the mechanisms of cryptic species coexistence in a nematode community through individual-based modelling. Oikos.

[ref-16] Danovaro R, Gambi C, Mirto S, Sandulli R, Ceccherelli VU (2004). Meiofauna. Biologia Marina Mediterranea.

[ref-17] De Meester N, Gingold R, Rigaux A, Derycke S, Moens T (2016). Cryptic diversity and ecosystem functioning: a complex tale of differential effects on decomposition. Oecologia.

[ref-18] De Troch M, Raes M, Muthumbi A, Gheerardyn H, Vanreusel A (2008). Spatial diversity of nematode and copepod genera of the coral degradation zone along the Kenyan coast, including a test for the use of higher-taxon surrogacy. African Journal of Marine Science.

[ref-19] Gad G (2009). Colonisation and speciation on seamounts, evidence from Draconematidae (Nematoda) of the Great Meteor Seamount. Marine Biodiversity.

[ref-20] Godfrey T (1996). Dive maldives. a guide to the maldives archipelago.

[ref-21] Hentschel U, Berger EC, Bright M, Felbeck H, Ott JA (1999). Metabolism of nitrogen and sulfur in ectosymbiotic bacteria of marine nematodes (Nematoda, Stilbonematinae). Marine Ecology Progress Series.

[ref-22] Hoegh-Guldberg O, Mumby PJ, Hooten AJ, Steneck RS, Greenfield P, Gomez E, Harvell CD, Sale PF, Edwards AJ, Caldeira K, Knowlton N, Eakin CM, Iglesias-prieto R, Muthiga N, Bradbury RH, Dubiand A, Hatziolos ME (2007). Coral reefs under rapid climate change and ocean acidification. Science.

[ref-23] Hong JH, Semprucci F, Raehyuk J, Kim K, Lee S, Jeon D, Yoo H, Kim J, Kim J, Yeom J, Lee S, Lee K, Lee W (2020). Meiobenthic nematodes in the assessment of the relative impact of human activities on coastal marine ecosystem. Environmental Monitoring and Assessment.

[ref-24] Ingels J, Valdes Y, Pontes LP, Silva AC, Neres PF, Corrêa GVV, Silver-Gorges I, Fuentes MMPB, Gillis A, Hooper L, Ware M, O’Reilly C, Bergman Q, Danyuk J, Sanchez Zarate S, Acevedo Natale LI, dos Santos GAP (2020). Meiofauna life on loggerhead sea turtles-diversely structured abundance and biodiversity hotspots that challenge the meiofauna paradox. Diversity.

[ref-25] Janßen A, Wizemann A, Klicpera A, Satari DY, Westphal H, Mann T (2017). Sediment composition and facies of coral reef Islands in the Spermonde Archipelago, Indonesia. Frontiers in Marine Science.

[ref-26] Jimenez H, Bigot L, Bourmaud C, Chabanet P, Gravier-Bonnet N, Hamel MA, Rilwan Y (2012). Multi-taxa coral reef community structure in relation to habitats in the Baa Atoll Man and Biosphere UNESCO Reserve (Maldives), and implications for its conservation. Journal of Sea Research.

[ref-27] Kang S, Ma W, Li FY, Zhang Q, Niu J, Ding Y, Han F, Sun X (2015). Functional redundancy instead of species redundancy determines community stability in a typical steppe of inner mongolia. PLOS ONE.

[ref-28] Lasagna R, Albertelli G, Colantoni P, Morri C, Bianchi CN (2010). Ecological stages of Maldivian reefs after the coral mass mortality of 1998. Facies.

[ref-29] Lawton JH, Brown VK, Schulze ED, Mooney HA (1993). Redundancy in ecosystems. Biodiversity and Ecosystem Function.

[ref-30] Loizeau JL, Arbouille D, Santiago S, Vernet JP (1994). Evaluation of a wide range laser diffraction grain size analyser for use with sediments. Sedimentology.

[ref-31] Loreau M, Naeem S, Inchausti P, Bengtsson J, Grime JP, Hector D, Hooperm U, Hustond A, Raffaelli B, Schmid D, Tilmanan D, Wardle A (2001). Biodiversity and ecosystem functioning: current knowledge and future challenges. Science.

[ref-32] Losi V, Grassi E, Balsamo M, Rocchi M, Gaozza L, Semprucci F (2021). Changes in taxonomic structure and functional traits of nematodes as tools in the assessment of port impact. Estuarine, Coastal and Shelf Science.

[ref-33] Losi V, Sbrocca C, Gatti G, Semprucci F, Rocchi M, Bianchi CN, Balsamo M (2018). Sessile macrobenthos (Ochrophyta) drives seasonal change of meiofaunal community structure on temperate rocky reefs. Marine Environmental Research.

[ref-34] Maria TF, Vanaverbeke J, Esteves AM, De Troch M, Vanreusel A (2012). The importance of biological interactions for vertical distribution of nematodes in a temperate ultra-dissipative sandy beach. Estuarine, Coastal and Shelf Science.

[ref-35] Montefalcone M, Morri C, Bianchi CN (2018). Long term change in bioconstruction potential of Maldivian coral reefs following extreme climate anomalies. Global Change Biology.

[ref-36] Montefalcone M, Morri C, Bianchi CN (2020). Influence of local pressures on Maldivian coral reef resilience following repeated bleaching events, and recovery perspectives. Frontiers in Marine Science.

[ref-37] Morgan KM, Kench PS (2016). Parrotfish erosion underpins reef growth, sand talus development and island building in the Maldives. Sedimentary Geology.

[ref-38] Morri C, Montefalcone M, Lasagna R, Gatti G, Rovere A, Parravicini V, Baldelli G, Colantoni P, Bianchi CN (2015). Through bleaching and tsunami: coral reef recovery in the Maldives. Marine Pollution Bulletin.

[ref-39] Ndaro SG, Olafsson E (1999). Soft-bottom fauna with emphasis on nematode assemblage structure in a tropical intertidal lagoon in Zanzibar, eastern Africa: I. Spatial variability. Hydrobiologia.

[ref-40] Nepote E, Bianchi CN, Chiantore M, Morri C, Montefalcone M (2016). Pattern and intensity of human impact on coral reefs depend on depth along the reef profile and on the descriptor adopted. Estuarine, Coastal and Shelf Science.

[ref-41] Neres PF, Dos Santos PJP, Esteves AM (2021). New chromadorid nematodes from Brazilian coral reefs: a taxonomic contribution to Chromadora Bastian, 1865. Zootaxa.

[ref-42] Netto SA, Attrill MJ, Warwick RM (1999a). The effect of a natural water-movement related disturbance on the structure of meiofauna and macrofauna communities in the intertidal sand flat of Rocas Atoll (NE, Brazil). Journal of Sea Research.

[ref-43] Netto SA, Warwick RM, Attrill MJ (1999b). Meiobenthic and macrobenthic community structure in carbonate sediments of Rocas Atoll (North-east, Brazil). Estuarine, Coastal and Shelf Science.

[ref-44] Pancrazi I, Ahmed H, Cerrano C, Montefalcone M (2020). Synergic effect of global thermal anomalies and local dredging activities on coral reefs of the Maldives. Marine Pollution Bulletin.

[ref-45] Petchey OL, Gaston KJ (2006). Functional diversity: back to basics and looking forward. Ecology Letters.

[ref-46] Raes M, De Troch M, Ndaro SGM, Muthumbi A, Guilini K, Vanreusel A (2007). The structuring role of microhabitat type in coral degradation zones: a case study with marine nematodes from Kenya and Zanzibar. Coral Reefs.

[ref-47] Raes M, Decraemer W, Vanreusel A (2008). Walking with worms: coral-associated epifaunal nematodes. Journal of Biogeography.

[ref-48] Raes M, Vanreusel A (2006). Microhabitat type determines the composition of nematode communities associated with sediment-clogged cold-water coral framework in the Porcupine Seabight (NE Atlantic). Deep Sea Research Part I: Oceanographic Research Papers.

[ref-49] Sahraeian N, Sahafi HH, Mosallanejad H, Ingels J, Semprucci F (2020). Temporal and spatial variability of free-living nematodes in a beach system characterized by domestic and industrial impacts (Bandar Abbas, Persian Gulf, Iran). Ecological Indicators.

[ref-50] Sandulli R, Semprucci F, Balsamo M (2014). Taxonomic and functional biodiversity variations of meiobenthic and nematode assemblages across an extreme environment: a study case in a Blue Hole cave. Italian Journal of Zoology.

[ref-51] Scharhauser F, Zimmermann J, Ott JA, Leisch N, Gruber-Vodicka HR (2020). Morphology of obligate ectosymbionts reveals Paralaxus gen. nov.: A new circumtropical genus of marine stilbonematine nematodes. Zoologica Scripta.

[ref-52] Schratzberger M, Somerfield PJ (2020). Effects of widespread human disturbances in the marine environment suggest a new agenda for meiofauna research is needed. Science of the Total Environment.

[ref-53] Schratzberger M, Warr K, Rogers SI (2007). Functional diversity of nematode community in the south-western North Sea. Marine Environmental Research.

[ref-54] Seinhorst JW (1959). A rapid method for the transfer of nematodes from fixative to anhydrous glycerine. Nematologica.

[ref-55] Semprucci F, Cesaroni L, Guidi L, Balsamo M (2018a). Do the morphological and functional traits of free-living marine nematodes mirror taxonomical diversity?. Marine Environmental Research.

[ref-56] Semprucci F, Colantoni P, Baldelli G, Rocchi M, Balsamo M (2010). The distribution of meiofauna on back-reef sandy platforms in the Maldives (Indian Ocean). Marine Ecology.

[ref-57] Semprucci F, Colantoni P, Baldelli G, Sbrocca C, Rocchi M, Balsamo M (2013). Meiofauna associated with coral sediments in the Maldivian subtidal habitats (Indian Ocean). Marine Biodiversity.

[ref-58] Semprucci F, Colantoni P, Balsamo M (2016). Is maturity index an efficient tool to assess the effects of the physical disturbance on the marine nematode assemblages? A critical interpretation of disturbance-induced maturity successions in some study cases in Maldives. Acta Oceanologica Sinica.

[ref-59] Semprucci F, Colantoni P, Sbrocca C, Baldelli G, Rocchi M, Balsamo M (2011). Meiofauna in sandy back-reef platforms differently exposed to the monsoons in the Maldives (Indian Ocean). Journal of Marine Systems.

[ref-60] Semprucci F, Frontalini F, Losi V, du Châtelet EA, Cesaroni L, Sandulli R, Balsamo M (2018b). Biodiversity and distribution of the meiofaunal community in the reef slopes of the Maldivian archipelago (Indian Ocean). Marine Environmental Research.

[ref-61] Semprucci F, Sbrocca C, Baldelli G, Tramontana M, Balsamo M (2017). Is meiofauna a good bioindicator of artificial reef impact?. Marine Biodiversity.

[ref-62] Sergeeva NG, Revkova TN (2021). First finding of Greeffiella Cobb, 1922 (Nematoda, Desmoscolecida) in the Black Sea. Ecologica Montenegrina.

[ref-63] Stoddart DR, Steers JA, Jones OA, Endean R (1977). The nature and origin of coral reef islands. Biology and Geology of Coral Reefs IV, Geology II.

[ref-64] Vanreusel A, Fonseca G, Danovaro R, Cristina da Silva M, Esteves AM, Ferrero T, Gad G, Galtsova V, Gambi MC, Da Fonsêca V, Ingels J, Ingole B, Lampadariou N, Merckx B, Miljutin D, Miljutina M, Muthumbi A, Netto S, Portnova D, Radziejewska T, Raes M, Tchesunov A, Vanaverbeke J, Van Gaever S, Venekey V, Bezerra TN, Flint H, Copley J, Pape E, Zeppilli D, Martinez PA, Galeron L (2010). The contribution of deep-sea macrohabitat heterogeneity to global nematode diversity. Marine Ecology.

[ref-65] Venekey V, Gheller PF, Maria TF, Brustolin MC, Kandratavicius N, Vieira DC, Brito S, Souza GS, Fonseca G (2014). The state of the art of Xyalidae (Nematoda, Monhysterida) with reference to the Brazilian records. Marine Biodiversity.

[ref-66] Vincx M, Hall GS (1996). Meiofauna in marine and freshwater sediments. Methods for the Examination of Organismal Diversity in Soils and Sediments.

[ref-67] Wieser W (1953). Die Beziehung zwischen Mundhöhlengestalt, Ernährungsweise und Vorkommen bei freilebenden marinen Nematoden. Arkiv för Zoologi.

[ref-68] Worm B, Barbier EB, Beaumont N, Duffy JE, Folke C, Halpern BS, Jackson JBC, Heike Lotze HK, Micheli F, Palumbi SR, Sala E, Selkoe KA, Stachowicz JJ, Watson R (2006). Impacts of biodiversity loss on ocean ecosystem services. Science.

[ref-69] Zeppilli D, Bongiorni L, Serrão Santos R, Vanreusel A (2014). Changes in nematode communities in different physiographic sites of the condor seamount (north-East atlantic ocean) and adjacent sediments. PLOS ONE.

